# Supplementary material for: Associations of Infant Feeding, Sleep, and Weight Gain with the Toddler Gut Microbiome
Source: Microorganisms. 2024 Mar 9;12(3):549. doi: 10.3390/microorganisms12030549 (PMC10972346; doi:10.3390/microorganisms12030549)
Supplement: Supplementary file 1 [file microorganisms-12-00549-s001.zip › microorganisms-2846453-supplementary.pdf]

Supplementary Table

Supplementary Table S1. Alpha diversity results for all feeding, sleeping and growth variables.

Testing Variable	Diversity Metric	Median (IQR)		H	p-value
		Category 1	Category 2		
Mode of birth	Shannon	4.64 (0.50)	4.76 (1.27)	0.335	0.563
Category 1: Vaginal	Observed OTUs	92 (43)	91 (53)	0.055	0.814
Category 2: C-section	FaithPD	9.22 (3.15)	7.96 (3.42)	1.424	0.233
	Evenness	0.73 (0.10)	0.73 (0.11)	0.633	0.426
Original study intervention	Shannon	4.55 (0.80)	4.82 (0.87)	0.366	0.545
Category 1: Control	Observed OTUs	82 (40)	103 (40)	2.193	0.139
Category 2: Intervention	FaithPD	7.72 (2.66)	9.80 (2.37)	2.955	0.086
	Evenness	0.73 (0.10)	0.72 (0.13)	0.025	0.874
Age of starting solids	Shannon	4.76 (0.87)	4.44 (1.45)	2.637	0.104
Category 1: Before 6 months	Observed OTUs	91 (43)	63 (45)	1.822	0.177
Category 2: 6 months and after	FaithPD	8.70 (2.94)	6.81 (3.40)	1.156	0.282
	Evenness	0.73 (0.12)	0.69 (0.16)	2.348	0.125
Any breastfeeding at 1 month	Shannon	4.55 (0.58)	4.76 (1.04)	0.404	0.525
Category 1: No	Observed OTUs	80 (37)	93 (39)	0.321	0.571
Category 2: Yes	FaithPD	8.55 (3.44)	8.70 (3.06)	0.024	0.877
	Evenness	0.71 (0.07)	0.73 (0.13)	0.248	0.619
Any breastfeeding at 6 months	Shannon	4.55 (0.55)	4.89 (1.28)	0.161	0.688
Category 1: No	Observed OTUs	91 (44)	86 (47)	0.136	0.712
Category 2: Yes	FaithPD	8.55 (3.20)	8.70 (3.54)	0.002	0.962
	Evenness	0.72 (0.10)	0.74 (0.15)	0.114	0.736
Any breastfeeding at 12 months	Shannon	4.65 (0.65)	4.76 (1.41)	0.117	0.732
Category 1: No	Observed OTUs	87 (45)	104 (55)	2.147	0.143
Category 2: Yes	FaithPD	8.46 (3.29)	9.84 (4.70)	2.202	0.138
	Evenness	0.73 (0.11)	0.69 (0.15)	0.036	0.849
Exclusive breastfeeding at 1 month	Shannon	4.75 (0.73)	4.53 (1.11)	0.123	0.726
Category 1: No	Observed OTUs	92 (45)	85 (45)	0.134	0.714
Category 2: Yes	FaithPD	8.78 (2.92)	8.55 (3.67)	0.001	0.975
	Evenness	0.73 (0.08)	0.70 (0.12)	0.328	0.567
Exclusive breastfeeding at 6 months	Shannon	4.65 (0.80)	4.72 (1.16)	0.125	0.724
Category 1: No	Observed OTUs	87 (43)	95 (58)	0.499	0.48
Category 2: Yes	FaithPD	8.47 (3.05)	9.06 (5.45)	0.08	0.778
	Evenness	0.72 (0.11)	0.74 (0.13)	0.125	0.724
Exclusive breastfeeding at 12 months	Shannon	4.65 (1.00)	4.76 (0.25)	0.076	0.782
Category 1: No	Observed OTUs	87 (46)	109 (23)	1.074	0.3
Category 2: Yes	FaithPD	8.62 (3.40)	10.07 (2.44)	0.577	0.447
	Evenness	0.73 (0.12)	0.71 (0.07)	0.019	0.89
Bed-sharing at 1 month	Shannon	4.54 (0.81)	5.05 (0.70)	4.335	0.037
Category 1: No	Observed OTUs	82 (43)	118 (23)	5.194	0.023
Category 2: Yes	FaithPD	8.36 (3.39)	9.98 (1.79)	3.409	0.065
	Evenness	0.72 (0.12)	0.76 (0.09)	2.228	0.135
Bedtime at 1 month	Shannon	4.89 (0.29)	4.55 (0.88)	0.324	0.569
Category 1: <21:00	Observed OTUs	97 (22)	86 (47)	0.081	0.776
Category 2: ≥21:00	FaithPD	8.99 (2.04)	8.70 (3.73)	0	1
	Evenness	0.73 (0.06)	0.73 (0.12)	0.155	0.694
Number of wakes at 1 month	Shannon	4.55 (0.91)	4.92 (0.83)	0.446	0.504
Category 1: ≤3	Observed OTUs	86 (42)	97 (38)	0.014	0.906
Category 2: >3	FaithPD	8.70 (2.94)	8.89 (3.37)	0.002	0.969
	Evenness	0.71 (0.13)	0.75 (0.04)	1.043	0.307
Max sleep bout at 1 month	Shannon	4.64 (0.69)	4.90 (0.87)	0.337	0.562
Category 1: ≤4 h	Observed OTUs	85 (52)	96 (36)	0.182	0.669
Category 2: >4 h	FaithPD	7.80 (3.85)	9.02 (1.32)	0.514	0.473
	Evenness	0.73 (0.12)	0.73 (0.15)	0.057	0.811
Night Sleep at 1 month	Shannon	4.90 (0.87)	4.54 (0.74)	2.228	0.135
Category 1: ≤10 h	Observed OTUs	96 (47)	86 (23)	0.347	0.556
Category 2: >10 h	FaithPD	9.02 (3.22)	8.25 (2.01)	0.5	0.48
	Evenness	0.74 (0.10)	0.67 (0.06)	3.125	0.077
Daytime sleep at 1 month	Shannon	4.53 (1.11)	4.81 (0.55)	1.95	0.163
Category 1: ≤8 h	Observed OTUs	83 (45)	103 (28)	1.951	0.162

Category 2: >8 h	FaithPD	8.62 (3.25)	9.42 (2.62)	0.963	0.326
	Evenness	0.72 (0.12)	0.73 (0.09)	2.167	0.141
Nap frequency at 1 month<br>Category 1: ≤4<br>Category 2: >4	Shannon	4.81 (0.70)	4.52 (1.06)	0.154	0.694
	Observed OTUs	86 (44)	107 (58)	1.581	0.209
	FaithPD	8.55 (2.75)	9.64 (5.31)	1.21	0.271
	Evenness	0.74 (0.08)	0.65 (0.11)	0.747	0.387
24hTST at 1 month<br>Category 1: ≤18 h<br>Category 2: >18 h	Shannon	4.55 (0.88)	4.75 (0.33)	0.048	0.827
	Observed OTUs	83 (46)	95 (34)	0.243	0.622
	FaithPD	9.00 (3.20)	8.46 (2.77)	0	1
	Evenness	0.74 (0.12)	0.71 (0.10)	0.001	0.971
Proportion of night to 24TST at 1 month<br>Category 1: ≤0.56<br>Category 2: >0.56	Shannon	4.92 (0.88)	4.47 (0.70)	4.395	0.036
	Observed OTUs	101 (48)	80 (17)	1.922	0.166
	FaithPD	9.23 (3.33)	7.96 (2.49)	2.444	0.118
	Evenness	0.75 (0.10)	0.69 (0.08)	4.102	0.043
Bedtime at 6 months<br>Category 1: <21:00<br>Category 2: ≥21:00	Shannon	4.99 (0.50)	4.54 (1.00)	1.986	0.159
	Observed OTUs	95 (42)	89 (46)	0.162	0.687
	FaithPD	9.37 (2.59)	8.47 (3.41)	0.253	0.615
	Evenness	0.78 (0.06)	0.71 (0.12)	2.759	0.097
Number of wakes at 6 months<br>Category 1: ≤1<br>Category 2: >1	Shannon	4.90 (0.87)	4.51 (0.92)	2.838	0.092
	Observed OTUs	98 (45)	82 (33)	1.298	0.255
	FaithPD	9.05 (3.17)	7.96 (3.01)	1.773	0.183
	Evenness	0.74 (0.08)	0.66 (0.10)	2.132	0.144
Max sleep bout at 6 months<br>Category 1: ≤7 h<br>Category 2: >7 h	Shannon	4.55 (0.69)	4.74 (0.88)	0.344	0.558
	Observed OTUs	91 (41)	83 (47)	0.274	0.601
	FaithPD	9.00 (3.47)	7.96 (3.34)	1.265	0.261
	Evenness	0.74 (0.13)	0.72 (0.12)	0.464	0.496
Night Sleep at 6 months<br>Category 1: ≤9 h<br>Category 2: >9 h	Shannon	4.54 (1.11)	4.81 (0.73)	0.002	0.962
	Observed OTUs	96 (46)	83 (31)	1.607	0.205
	FaithPD	9.02 (2.85)	7.64 (3.11)	1.988	0.159
	Evenness	0.73 (0.12)	0.73 (0.09)	0.183	0.669
Daytime sleep at 6 months<br>Category 1: ≤5 h<br>Category 2: >5 h	Shannon	4.55 (0.95)	4.74 (0.79)	0.892	0.345
	Observed OTUs	83 (45)	115 (34)	4.248	0.039
	FaithPD	7.48 (3.34)	9.87 (1.88)	8.621	0.003
	Evenness	0.73 (0.12)	0.70 (0.15)	0.05	0.823
Nap frequency at 6 months<br>Category 1: ≤3<br>Category 2: >3	Shannon	4.76 (0.90)	4.51 (1.04)	2.031	0.154
	Observed OTUs	91 (45)	82 (48)	0.382	0.536
	FaithPD	8.40 (3.24)	9.00 (3.83)	0.184	0.668
	Evenness	0.74 (0.09)	0.65 (0.09)	3.128	0.077
24hTST at 6 months<br>Category 1: ≤13 h<br>Category 2: >13 h	Shannon	4.53 (0.92)	4.81 (0.73)	0.405	0.524
	Observed OTUs	82 (35)	106 (38)	2.535	0.111
	FaithPD	7.56 (2.56)	9.58 (1.80)	4.55	0.033
	Evenness	0.74 (0.11)	0.71 (0.11)	0.146	0.702
Proportion of night to 24TST at 6 months<br>Category 1: ≤0.66<br>Category 2: >0.66	Shannon	4.65 (0.66)	4.68 (1.05)	0.626	0.429
	Observed OTUs	107 (38)	76 (40)	5.559	0.018
	FaithPD	9.46 (2.46)	6.90 (3.08)	11.676	0.001
	Evenness	0.72 (0.13)	0.73 (0.12)	0.016	0.899
Bedtime at 12 months<br>Category 1: <21:00<br>Category 2: ≥21:00	Shannon	5.18 (0.38)	4.54 (0.94)	4.054	0.044
	Observed OTUs	121 (14)	83 (45)	4.797	0.029
	FaithPD	10.32 (2.16)	8.38 (3.43)	3.854	0.05
	Evenness	0.75 (0.04)	0.71 (0.12)	1.713	0.191
Number of wakes at 12 months<br>Category 1: ≤1<br>Category 2: >1	Shannon	4.55 (0.59)	4.81 (1.37)	0.003	0.959
	Observed OTUs	93 (47)	82 (43)	0.404	0.525
	FaithPD	8.70 (2.71)	8.40 (4.19)	0.215	0.643
	Evenness	0.73 (0.09)	0.73 (0.15)	0.05	0.823
Max sleep bout at 12 months<br>Category 1: ≤9 h<br>Category 2: >9 h	Shannon	4.89 (0.87)	4.47 (0.90)	0.779	0.378
	Observed OTUs	91 (37)	80 (53)	0.644	0.422
	FaithPD	9.00 (3.22)	7.96 (3.63)	1.448	0.229
	Evenness	0.73 (0.12)	0.72 (0.11)	0.161	0.688
Night Sleep at 12 months<br>Category 1: ≤10 h<br>Category 2: >10 h	Shannon	4.65 (1.11)	4.65 (0.63)	0.305	0.581
	Observed OTUs	83 (47)	116 (21)	1.127	0.288
	FaithPD	8.38 (3.99)	9.35 (1.14)	0.795	0.373
	Evenness	0.73 (0.13)	0.71 (0.06)	0.002	0.966
Daytime sleep at 12 months<br>Category 1: ≤3 h<br>Category 2: >3 h	Shannon	4.49 (1.28)	4.75 (0.52)	0.557	0.455
	Observed OTUs	83 (52)	95 (36)	0.51	0.475
	FaithPD	8.02 (3.39)	9.01 (2.53)	1.521	0.218
	Evenness	0.72 (0.13)	0.73 (0.05)	0.237	0.626

Nap frequency at 12 months<br>Category 1: ≤2<br>Category 2: >2	Shannon	4.55 (0.99)	4.76 (0.75)	1.498	0.221
	Observed OTUs	83 (44)	96 (41)	1.683	0.195
	FaithPD	8.55 (3.14)	9.00 (2.63)	0.457	0.499
	Evenness	0.72 (0.12)	0.75 (0.15)	1.682	0.195
24hTST at 12 months<br>Category 1: ≤13 h<br>Category 2: >13 h	Shannon	4.49 (1.08)	4.75 (0.56)	1.147	0.284
	Observed OTUs	83 (51)	95 (38)	0.607	0.436
	FaithPD	8.02 (3.41)	9.01 (2.53)	1.29	0.256
	Evenness	0.72 (0.13)	0.73 (0.09)	0.886	0.347
Proportion of night to 24TST at 12 months<br>Category 1: ≤0.77<br>Category 2: >0.77	Shannon	4.55 (0.76)	4.91 (1.06)	1.038	0.308
	Observed OTUs	87 (41)	95 (41)	0.329	0.567
	FaithPD	8.47 (3.47)	8.87 (3.17)	0.016	0.899
	Evenness	0.71 (0.10)	0.75 (0.11)	1.464	0.226
RWG - Birth to 6 months<br>Category 1: No<br>Category 2: Yes	Shannon	4.81 (0.63)	4.41 (0.87)	2.55	0.11
	Observed OTUs	96 (39)	72 (34)	2.95	0.086
	FaithPD	9.00 (3.11)	6.81 (3.31)	3.633	0.057
	Evenness	0.73 (0.12)	0.72 (0.11)	0.958	0.328
